# British Institutions for the Care of the Inebriate—IV

**Published:** 1903-11-21

**Authors:** 


### HOSPITAL ADMINISTRATION.

### CONSTRUCTION AND ECONOMICS.

### BRITISH INSTITUTIONS FOR THE CARE OF THE INEBRIATE.—IV.

By Our Special Medical Commissioner.

(*Continued from Page* 72)

THE NORWOOD SANATORIUM FOR THE TREATMENT OF INEBRIETY.

The customary institutional treatment by restraint is, it must be admitted, beset by difficulties. It is irksome, expensive, and oftentimes very uncertain in its results. The best opinion favours the method of treating an inebriate "under the Act" and enforcing strict abstinence, a regular hygienic existence, and strict compliance with moral and social requirements until time and discipline shall have removed, as far as possible, the damage done to the physical basis and allowed of the upbuilding of sound textures from which morbid psychological manifestations no longer arise. Unfortunately, in many instances, even after long periods of "detention," the tendency to inebriety speedily returns or indeed is never overcome, and the advantages of "isolation" from alcoholics are thus quickly lost.

But where the opportunity for obtaining compulsory abstinence over a long period cannot be arranged, it is claimed that much advantage may oftentimes be secured by treatment extending over a comparatively short period.

The opinion of such an expert in the study of inebriety as Dr. Welsh Branthwaite, H.M. Inspector of Retreats, is particularly valuable in regard to this matter: "There is no royal road to 'cure' for the confirmed inebriate, he has to be broken from his habit, brought back to physical health and taught by moral influence to live his life without resort to alcohol in any shape or form. I am quite in accord with those who insist, as a first principle, upon the value of long-continued enforced abstinence; and I am inclined to agree with the licensees of some retreats who, as a matter of principle, decline to accept any patient for treatment who will not consent to a term of detention extending to twelve months or over. There is no doubt whatever that the longer residence cases do better than those of shorter terms. It is, however, undesirable that *every* institution shall make a hard and fast line not to admit short term cases. There are many inebriates, especially men, who are tied to their occupation and cannot afford to retire into seclusion for long periods. It is such persons, finding themselves blocked from treatment in a recognised institution by the necessity of signing for impossible months, who are driven to resort to 'cures' which promise recovery in three or four weeks. I cannot help thinking that some good results might be obtained amongst persons who could manage to undergo control and treatment for short periods only. I am not prepared to advocate the admixture in one retreat of short with long term cases, but I do think, especially near London, that there is a great demand for an institution entirely devoted to the reception of patients able and willing to sign for a month, or even less. Such an establishment should more closely approximate to a hospital than to an ordinary retreat of the 'home' type, and should be designed for, and be prepared to receive at the shortest notice, the most acute type of cases. Although the percentage of permanent good results would necessarily prove smaller than the long-period homes produce, still a retreat such as I suggest would at least afford a chance of recovery to many who are at present debarred by commercial and other ties from the benefits of longer control. It is possible also that many patients having tried the shorter periods and experienced failure would, when circumstances permit, willingly consent to more extended treatment." *

Dr. C. A. McBride has organised a method of managing inebriates and patients addicted to drug indulgence by what he is pleased to term "The Intensity Treatment," which, by "a short period" procedure of some six weeks, is supposed to arrive at a satisfactory "cure." Dr. McBride's establishment, formerly known as the Dell, but now termed "Norwood Sanatorium," is situate at 93 Church Road, Upper Norwood, S.E. The house is an old mansion delightfully situated in charmingly wooded grounds with sloping lawns, and having an extended view rich in varied attractions. The site is one of the best on the south side of London, and the proximity of the Crystal Palace is also said to be advantageous for the patients.

Detention and restraint are apparently not employed. In order to prevent any misconception we quote from a booklet furnished us by Dr. McBride: "Since the treatment is curative and not repressive, it is found quite unnecessary to enforce those irksome conditions of restraint which prevent so many who ought to be patients from submitting to what appears to them as something very like imprisonment. It is of course necessary for patients to present themselves for treatment at the three times requisite in the course of the day and to observe the directions of the medical superintendent in other respects, but otherwise they can dispose of their time as they will within the ordinary limits of reason." It is seen at once that such procedure differs radically from that customary in the majority of retreats.

Dr. McBride claims that his results are eminently satisfactory. We regret that hitherto we have had no opportunity of submitting such contention to impartial investigation. Much reliance is placed in the use of medicinal agents. We consider it a serious omission that no full description of the treatment has as yet been published, but Dr. McBride assures us that "the detailed account of the treatment and the various drugs employed will be given in my book on the modern treatment of alcoholism, now in course of preparation"; and which, needless to say, we shall be prepared to submit to an unprejudiced examination. As far as we can at present gather, the subcutaneous administration of strychnine and other powerful drugs plays an important part in the treatment. Much is done to restore tone to the enfeebled nervous system, careful regulation of the nutritional processes is secured, and the excretory functions are stimulated.

The advantages claimed for the so-called "intensity treatment," which we venture to think is an ambiguous and not altogether scientific or desirable designation, are certainly very great, indeed the practitioner experienced in the customary moral and medical methods of management will, we imagine, be likely to remain sceptical regarding them—"absolutely no restraint and therefore no demoralisation,

* "The Report of the Inspector under the Inebriates Acts, 1879 to 1900, for the year 1901." London: Eyre and Spottiswoode. 1902. P. 15.

the charm of a natural and home-like life, the daily temptation similar to that which the patient will have to meet on returning home, the short time, viz., six weeks, the absolute removal of all craving for stimulants, so that neither illness, suffering, worry, depression, nor weakness can possibly reproduce the craving, the permanent results and the ultimate saving of expense." It is rather remarkable, however, that "its one weak point is its unsuitability in cases where vice is the predominating factor."

We fear most will consider that the claims are such as experience will hardly be likely to uphold, but since it is contended that much benefit has accrued from the treatment advocated, as Dr. McBride well says, "it remains for those who are interested to look into the matter for themselves, and weigh the evidence which is offered them."

The nearest railway stations to the sanatorium are those at the Crystal Palace, and known as the High and Low Level. The former is ten minutes' walk from the Home, the latter about fifteen minutes.

*To be continued.*

